# Supplementary material for: Modeling the iron storage protein ferritin reveals how residual ferrihydrite iron determines initial ferritin iron sequestration kinetics
Source: PLoS One. 2023 Feb 6;18(2):e0281401. doi: 10.1371/journal.pone.0281401 (PMC9901743; doi:10.1371/journal.pone.0281401)
Supplement: S1 File — File contains complete set of model equations. (PDF) [file pone.0281401.s001.pdf]

$$\frac{\mathrm{d}[\mathrm{LIP}]}{\mathrm{d}t} = -2 \cdot \frac{\mathrm{kcat}_{(\mathrm{Fe\ oxidation})} \cdot \frac{H + \mathrm{rO}}{24 + \mathrm{rO}} \cdot [\mathrm{FT\text{-}cage}] \cdot [\mathrm{LIP}]^{n_{(\mathrm{Fe\ oxidation})}}}{\mathrm{Km}_{(\mathrm{Fe\ oxidation})}{}^{n_{(\mathrm{Fe\ oxidation})}} + [\mathrm{LIP}]^{n_{(\mathrm{Fe\ oxidation})}}}$$

$$+ 2 \cdot \mathrm{k1}_{(\text{"Fe reduction"})} \cdot [\mathrm{DFP}] + \mathrm{k}_{\mathrm{FTlysis}} \cdot [\mathrm{core}]$$

$$\frac{\mathrm{d}[\mathrm{FT\text{-}cage}]}{\mathrm{d}t} = -\mathrm{k}_{\mathrm{FTlysis}} \cdot [\mathrm{FT\text{-}cage}] + v_{(\mathrm{FT\ expression})}$$

$$\frac{\mathrm{d}[\mathrm{core}]}{\mathrm{d}t} = 2 \cdot \frac{\mathrm{k1}_{(\mathrm{core\ formation})} \cdot [\mathrm{DFP}] \cdot [\mathrm{core}]}{\mathrm{k2}_{(}\mathrm{core\ formation)} + [\mathrm{DFP}]} \cdot \frac{\mathrm{k3}_{(\mathrm{core\ formation})}{}^{n_{(\mathrm{core\ formation})}}}{\mathrm{k3}_{(\mathrm{core\ formation})}{}^{n_{(\mathrm{core\ formation})}} + [\mathrm{core}]^{n_{(\mathrm{core\ formation})}}}$$

$$\cdot \frac{4300^{m_{(\mathrm{core\ formation})}} - \mathrm{atoms\ per\ cage}^{m_{(\mathrm{core\ formation})}}}{4300^{m_{(\mathrm{core\ formation})}}}$$

$$+ 4 \cdot \mathrm{k1}_{(\mathrm{nucleation})} \cdot [\mathrm{DFP}]^2 \cdot [\mathrm{FT\text{-}cage}] \cdot \frac{L + \mathrm{rN}}{24 + \mathrm{rN}} \cdot \frac{\mathrm{ki}_{(\mathrm{nucleation})}{}^{n_{(\mathrm{nucleation})}}}{\mathrm{ki}_{(\mathrm{nucleation})}{}^{n_{(\mathrm{nucleation})}} + [\mathrm{core}]^{n_{(\mathrm{nucleation})}}}$$

$$- \mathrm{k}_{\mathrm{FTlysis}} \cdot [\mathrm{core}]$$

$$\frac{\mathrm{d}[\mathrm{DFP}]}{\mathrm{d}t} = \frac{\mathrm{kcat}_{(\mathrm{Fe\ oxidation})} \cdot \frac{H + \mathrm{rO}}{24 + \mathrm{rO}} \cdot [\mathrm{FT\text{-}cage}] \cdot [\mathrm{LIP}]^{n_{(\mathrm{Fe\ oxidation})}}}{\mathrm{Km}_{(\mathrm{Fe\ oxidation})}{}^{n_{(\mathrm{Fe\ oxidation})}} + [\mathrm{LIP}]^{n_{(\mathrm{Fe\ oxidation})}}}$$

$$- \frac{\mathrm{k1}_{(\mathrm{core\ formation})} \cdot [\mathrm{DFP}] \cdot [\mathrm{core}]}{\mathrm{k2}_{(\mathrm{core\ formation})} + [\mathrm{DFP}]} \cdot \frac{\mathrm{k3}_{(\mathrm{core\ formation})}{}^{n_{(\mathrm{core\ formation})}}}{\mathrm{k3}_{(\mathrm{core\ formation})}{}^{n_{(\mathrm{core\ formation})}} + [\mathrm{core}]^{n_{(\mathrm{core\ formation})}}}$$

$$\cdot \frac{4300^{m_{(\mathrm{core\ formation})}} - \mathrm{atoms\ per\ cage}^{m_{(\mathrm{core\ formation})}}}{4300^{m_{(\mathrm{core\ formation})}}}$$

$$- \mathrm{k1}_{(\text{"Fe reduction"})} \cdot [\mathrm{DFP}]$$

$$- 2 \cdot \mathrm{k1}_{(\mathrm{nucleation})} \cdot [\mathrm{DFP}]^2 \cdot [\mathrm{FT\text{-}cage}] \cdot \frac{L + \mathrm{rN}}{24 + \mathrm{rN}} \cdot \frac{\mathrm{ki}_{(\mathrm{nucleation})}{}^{n_{(\mathrm{nucleation})}}}{\mathrm{ki}_{(\mathrm{nucleation})}{}^{n_{(\mathrm{nucleation})}} + [\mathrm{core}]^{n_{(\mathrm{nucleation})}}}$$

$$\mathrm{atoms\ per\ cage} = \frac{[\mathrm{core}]}{[\mathrm{FT\text{-}cage}]}$$

$$L = 24 - H$$
