# Supplementary material for: Modeling the iron storage protein ferritin reveals how residual ferrihydrite iron determines initial ferritin iron sequestration kinetics
Source: PLoS One. 2023 Feb 6;18(2):e0281401. doi: 10.1371/journal.pone.0281401 (PMC9901743; doi:10.1371/journal.pone.0281401)
Supplement: S2 File — Zip file containing all model files and considerations for adding the FT sub model to a cell model. (ZIP) [file pone.0281401.s002.zip › Considerations for adding FT submodel to cell.docx]

# References

1. Crowe PT, Marsh MN. Morphometric analysis of small intestinal mucosa. IV. Determining cell volumes. *Virchows Arch A Pathol Anat Histopathol*. 1993;422(6):459-466. doi:10.1007/BF01606454

2. MacLeod RJ, Hamilton JR, Bateman A, et al. Corticostatic peptides cause nifedipine-sensitive volume reduction in jejunal villus enterocytes. *Proc Natl Acad Sci U S A*. 1991;88(2):552-556.

## Considerations for adding FT submodel to cell

We designed the model so one could make several adjustments to it when incorporating it into a cellular model of iron metabolism in a particular cell type. The first thing that can be adjusted is the cell volume. We used the estimated volume of an enterocyte with a value of 1.4e-12 L [1,2, 55]. Next is the FT concentration. There is a range of concentrations hypothesized for FT, with particular cell types having different concentrations. Next is the subunit composition. FT has 24 subunits that could be of 2 types, the heavy (H) and light (L) that have different functions. The H subunit contains a ferroxidase and impacts the oxidation reaction, while L is hypothesized to serve some role in nucleation, catalyzing mineralization from DFP. To accommodate the fact that cells can vary the H:L ratio we have provided H and L as global variables, so that their values can change during a simulation if needed.

The extensibility of the model goes beyond the above highlighting and prediction of necessary ports by which to modify the model for a particular purpose. Specifically, this model is packaged to meet the model brick specification. This includes deposition on the BioModels Database [12] in the SBML format (MODEL2211030001).
