# Supplementary material for: Modeling the iron storage protein ferritin reveals how residual ferrihydrite iron determines initial ferritin iron sequestration kinetics
Source: PLoS One. 2023 Feb 6;18(2):e0281401. doi: 10.1371/journal.pone.0281401 (PMC9901743; doi:10.1371/journal.pone.0281401)
Supplement: Attachment — Submitted filename: Response to Reviewers.pdf [file pone.0281401.s004.pdf]

UCONN HEALTH
Richard D. Berlin Center for
Cell Analysis and Modeling

Detailed answers to Reviewers' Comments:

We would like to thank all reviewers for their constructive critiques which have allowed us to improve the manuscript. Here are detailed responses to their points:

Reviewer #1:

1. **The abstract is quite disconnected from the introduction. The abstract majorly focused on the iron metabolism and the intro starts with the modeling. There should be a better way to present this work to make its flow more consistent.**

We agree and have revised the abstract to focus more on modeling and mirror the introduction to make its flow more consistent.

2. **In figure 2 which is the calibration and validation process, the predictions seem quite different from the real data. Could the authors add some statistical measurement to show how close their prediction is to the real data?**

The sum of squares of weighted residuals is usually how such a distance between predictions and data is expressed (and is indeed the objective function for parameter estimation). However this metric is meaningless per se given that it depends on the magnitude of the measured variables and the weights. Thus, while we can state the actual value here (0.849), there is no way to assess if that number is high or low on its own. This is why displaying the data versus model (as in Fig 2) is the best way to assess the fit.

We also carried out one more test (suggested by comments of reviewer 3), which is to allow the estimated parameter values to change within +/-10% of the fitted value and ask whether a better fit to the data could be obtained. This is now shown in Fig 2e. Note though, that while overall the model of Fig 2e is a better fit to the data, the model of Fig 2d manages to fit the data after 20

seconds much better than Fig 2e. The initial few seconds of this experiment correspond to apoferritin or ferritin with very low levels of internalized iron. Since our model is intended to be used for cellular models, we think that it is more important to fit ferritin's behavior at longer time scales, than the short time scales of *in vitro* experiments with apoferritin (as is the case with the experiment of Figs 2d and 2e). We also noted that when we applied the parameter values of Fig 2e to the data of Fig 2b and 2c we would obtain much worse validation there.

3. **The authors stated that including the subunit composition is one of the highlights for this paper. So is there any situations the cells or human adjust the ratio of H-subunit to L-subunit to change the ferritin composition? And can your data match those conditions?**

This is a great suggestion. It allowed us to revise many areas in the text to reflect the advantage of including subunit effects and where the manuscript was not describing important details:

- Most importantly, the model was calibrated and validated against experimental data that used FT with different subunit compositions. The subunit composition was changed to reflect the experimental conditions for each separate calibration and validation simulation. We have updated the legend of Fig 2 to reflect this.
- We added tissue-specific subunit expression to the "Introduction" section.
- We added the sentence "Additionally, we were also able to include subunit detail with the model developed here, and set the "H" parameter to 4 to reflect what is known about human hepatocyte FT subunit composition" to that section to inform the reader of the possibility of changing subunit composition based on cell cell type.
- We added discussion in the subunit subsection of the "Results" about the qualitative match in data between our results and published data on heteropolymers.
- Lastly we'd like to point out that we discuss how the data informed the parameters rO and rN in the model ("Model Definition" section) (Carmonal et al. 2014, Bradley et al. 2014, Finazzi et al. 2014, refs 44, 47, 48). To change the impact of H and L on oxidation and nucleation, it is possible to calibrate the model to a particular system by changing the rO and rN parameters.

Reviewer #2:

1. **There are some reports that copper and other metals can also be stored by ferritin. What is the take on that by the authors? Since iron sequestration determines the sequestration kinetics, how do other cations influence the kinetics? Could the authors model with concentrations and crowding present in cells, or at least comment and discuss this?**

We agree, though the quantitative extent of this may be limited (particularly in the case of copper which is a metal existing at a much lower concentration than iron in the body). Our model is developed so that it would be possible to expand to include incorporation of other metals into the core, so the present model could be used as a building block for that too. As such, we expanded the discussion to touch upon our model's intended purpose and how it could be expanded.

2. **A recent review greatly summarized the chemistry and biology of ferritin, including the formation of the polymers and discussing the different models of iron sequestration known. Please cite it here (Metallomics. 2021 May 12;13(5):mfab021. doi: 10.1093/mtomcs/mfab021).**

We are grateful for bringing this paper to our attention. It is now citation number 17 in the manuscript.

3. **FT can have different ratios of FTH and FTL. How does this influence the proposed model by the authors?**

This is a great observation. Our model addresses this by including a parameter "H" and a parameter "L". These represent the number of each type of subunit with the 24mer and affect the oxidation and nucleation rate laws as a consequence of their experimentally determined roles. The H and L subunits are discussed specifically in how they affect the oxidation and nucleation reactions of the model in the "Model Definition" section, and in response to comment 3 by reviewer 1. Parameters H and L allow a modeler to adjust this model to reflect the appropriate H/L ratio for specific ferritin

Cell and Genome Sciences Building
400 Farmington Avenue
Farmington, CT 06030-6406
PHONE 860.679.1452 · **FAX** 860.679.1030

Richard D. Berlin
Center for cell analysis and modeling
health.uconn.edu/cell-analysis-modeling/
pmendes@uchc.edu

compositions. This is indeed what we did in the section on hepatocyte iron model (where we set H to 4 and L to 20)

4. **Can the authors comment if their model can be transferred to mitochondrial ferritin? This should be at least discussed.**

Mitochondrial ferritin has its own specific subunit, which has higher similarity to cytoplasmic ferritin H than to L. This is a great suggestion, in line with comment 1 by reviewer 2. Along a similar vein we added: "An example of tuning this model for a specialized case would be to represent mitochondrial ferritin (MtF). MtF is a homopolymer of 24 subunits that have a high degree of sequence homology with human H-chain ferritin (HuHF). Thus, the model with the H parameter set to 24 could serve as a starting point, but it may be better to adjust the parameter values specifically to MtF data for a more accurate model. Indeed, MtF and HuHF have been shown to have striking differences in their ferroxidase activity, despite their high level of homology." to the "Discussion" section.

Reviewer #3:

1. **The authors present a model that attempts to describe the kinetics of iron mineralization in ferritin by incorporating the essential features of Fe(II) transport, binding, oxidation, nucleation and mineralization. Unfortunately, the model neglects several features that have been identified in experiments, including 3-site binding at the ferroxidase site, with binding at the third site helping to dislodge the di-iron DFP from the ferroxidase site; the option for iron oxidation and subsequent mineralization occurring directly at the mineral surface; the transient role of the diferric peroxo intermediate, the role of the mu-oxo diferric intermediate, and the role of water deprotonation in the mineralization process**

We are grateful for this comment as it showed that we had not properly justified the level of detail intended for this model. Indeed, mechanistically a lot is known about how an individual FT 24mer sequesters iron, revealed by *in vitro* experiments. There is no doubt that the details mentioned by

Cell and Genome Sciences Building
400 Farmington Avenue
Farmington, CT 06030-6406
PHONE 860.679.1452 · **FAX** 860.679.1030

Richard D. Berlin
Center for cell analysis and modeling
health.uconn.edu/cell-analysis-modeling/
pmendes@uchc.edu

the reviewer would be essential to include in a model of FT iron sequestration that would be intended to reflect all the mechanistic details known. Our goal though, was to produce a model that is able to capture the behavior of ferritin in a cellular context where it will be accompanied by many other cellular processes. Therefore, one of the important objectives of this model is that it should have a small number of species and reactions, while still trying to represent the observed behavior of ferritin. As with all models, here the critical aspect is how much detail to capture versus how complex the model has to be to do so. As mentioned in the answer 2 to Reviewer 1, when the model cannot represent aspects that are mostly observed *in vitro* rather than *in vivo*, we opt for the model to better represent the latter (see the case of Fig 2d and 2e).

We have now added a paragraph describing the scope and purpose of the model more clearly in the discussion section. We also revised the abstract to better frame the manuscript overall. We do avoid claiming in the manuscript that this model would be fully reflective of the known detailed mechanisms of ferritin, and stress that its main purpose is to become a component of cellular models of iron metabolism.

2. **In the common H:L ferritin heteropolymers, it makes sense that most or all L ferritins could be engaged in nucleating mineral formation, which would help to spread the mineral more broadly over the protein surface and allow for more complete filling of the protein cavity. The authors instead advance the hypothesis that only a small number of nucleation sites are involved, without providing evidence.**

We do not advance that hypothesis. We did cite the electron microscopy works of Narayanan et al. 2019 and López-Castro JD et al. 2012 (refs 24 and 45) which suggest that, however, our model does not depend on it. The model is only temporal and has no spatial discrimination of the core. Thus it cannot represent more or less spread out locations of the nucleation events. Instead the model represents nucleation and mineralization as specific reactions with rates. The rate of nucleation increases with the proportion of L subunits, which we believe is in line with what the reviewer suggests, namely “that most or all L ferritins could be engaged in nucleating mineral formation”.

Richard D. Berlin Center for
Cell Analysis and Modeling

3. **The authors' models do not agree particularly well with the experimental data in figure 2, in particular Figs 2C and 2E. Little justification is given for these discrepancies, but they imply deficiencies in the model.**

Indeed this issue needed better discussion, thank you.

We had originally included a comment that reflects the possibility of some experimental artifacts (due to being possible that "apoferritin" in experiments could already have a small number of iron atoms incorporated): "The influence of a few residual iron atoms is not accounted for when running the model is one possible explanation for why in Fig 2C the validation steady state seems to be a good fit but the initial part of the simulated time course deviates from the experimental observations; the model assumes 0 initial iron inside FT but if that is not the case in the experimental setting the model would not be accurate (i.e. even if only very low amounts of sequestered iron are present experimentally). In fact, when the APC is increased to between 75-100 in the model, the fit to the data is improved."

We further explored this issue, investigating if allowing small variations of the parameter values could make the model better approximate the experimental data of the original Fig 2c. We have now included a panel 2e in the revised version which shows the best approximation to the data if the parameter values are allowed to change with +/-10% of their original values. As is evident in Fig 2e the overall fit is better, particularly in the early time (low amounts of incorporated iron), but the behavior at times larger than 20 seconds (for larger amounts of incorporated iron) the parameters of Fig 2d are actually better. Given that apoferritin is very short-lived *in vivo* (only appearing after increases in protein levels of FTH1 and FTL), we therefore think that the parameters of Fig 2d are still better for our model's purpose. Additionally with the parameter values of Fig 2e the fits in Fig 2b and 2c would become much worse.

4. **If understanding biology is the goal, it seems more realistic to consider mineralization kinetics under conditions of low levels of free iron. Is the addition of thousands of equivalents of Fe(II) per ferritin 24mer relevant to physiologic conditions?**

We fully agree that it is vital to capture a physiologic range of free iron levels. The model is validated by a large range of iron levels, including equivalents in the 10-100 range (Fig 2) as well as >1000 (Fig 4c). We are grateful to have been pointed to (J. Mol. Biol. (2005) 352, 467–477) which

uses 0-20 Fe per cage to validate against as well. The results of the reproduction are also relevant and shown in reference to comment 6 below. Also, in the Results section, the FT and LIP concentrations were explicitly chosen to represent physiologically relevant values.

5. **In the physiologic scenario, there is presumably rarely an empty "apoferritin", and most mineralization must occur at partially filled ferritin cavities. Understanding the details of how partially filled ferritin mineralizes additional free iron is important to model accurately.**

We agree fully. This is why we stated in the Discussion that "the APC effect and the lack of experiments where APC is larger than a few atoms suggests the effect of this variable should be studied experimentally much more in depth". Unfortunately there is a scarcity in experimental data outside of apoferritin, even the suggested publication (J. Mol. Biol. (2005) 352, 467–477) relies on apoHoSF. This is why it was so important for us to calibrate the model with the data in Harrison *et al.* 1974, ref 40, where a full range of FT core contents were followed experimentally. This is also why we devote an entire section of our Results on the "Effect of pre-existing iron core on FT iron sequestration dynamics" and simulate the full range (0 - 4300 atoms) in multiple figures (Fig 3 and 6).

6. **There is strong experimental evidence that under low- or high-iron-loading conditions in ferritin heteropolymers, the ferroxidase centers control chemical reactivity and generate DFP species only at initial stages of mineralization. (J. Mol. Biol. (2005) 352, 467–477). At subsequent stages, mineralization can be modeled to occur mostly at the mineral core via an autocatalytic mechanism.**

We are grateful for reviewer 3 pointing us to the above publication. The paper presents a kinetic model that we can use as additional validation of our model's behavior. The determination of the transience in ferric intermediate formation before mineralization via an autocatalytic mechanism is robustly shown in the experimental data. Because our model also calculates the formation of a peroxo intermediate (DFP) and includes an autocatalytic mineralization reaction we sought to assess how well it could reproduce such results — critically without allowing a fluctuation greater than 10% in the model parameters. When we plot a time course of FT iron sequestration in our model,

we see identical results, DFP forms initially but then additional iron is predominantly added to the core.

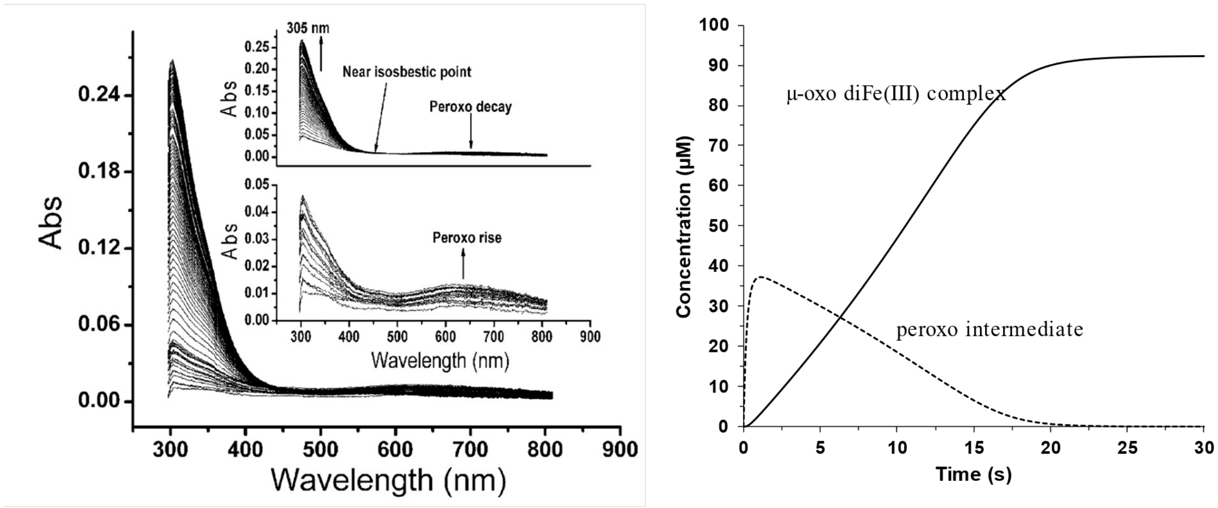

Fig 1. Conditions: 14 μM apo-HoSF in 0.1 M Mops (pH 7.0) saturated with 100% O2, 92.4 μM Fe(II), 25 °C

Our model qualitatively recapitulates the transience in DFP formation.as shown in the above figure, in our model there is an initial formation of DFP after Fe addition, but as mineralization proceeds, there is a peroxo decay as in the experimental data.

In addition to addressing the DFP transience, because of the credibility of the experimental data in the recommended publication, we also sought to reproduce an additional observation from it to further validate the model. The results from running the model are shown below side by side with the experimental data and now in Fig 2g.

Richard D. Berlin Center for
Cell Analysis and Modeling

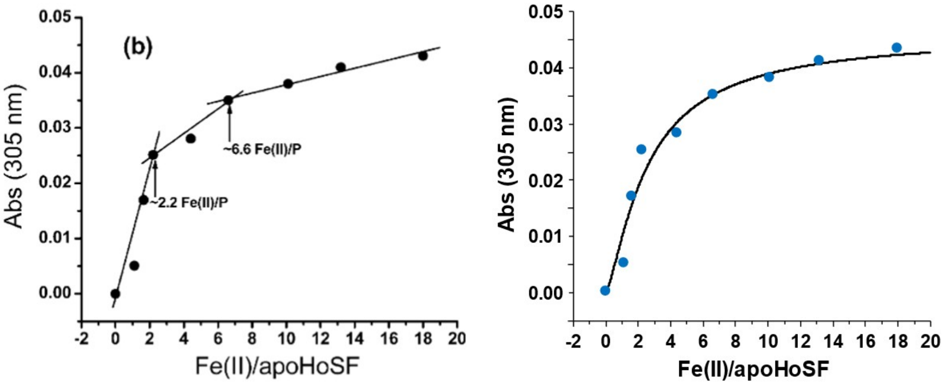

Fig 2. Absorbance at (b) 305 nm at 150 ms versus iron loading of HoSF. Conditions: 22.0 µM HoSF in 0.1 M Mops (pH 7.0) saturated with 100% O2, 24.2–145.2 µM $FeSO_4$, 25 ºC.

7. **The authors do not appear to take the available experimental data for heteropolymers into account in developing their own model. It may be too challenging, confusing and generally not useful to compare pure H homopolymer to H+L heteropolymers in this work, based on what are likely to be very different mineralization mechanisms. The authors unfortunately gloss over these differences despite considerable work by Chasteen, Theil and others comparing homo- vs. heteropolymer biomineralization.**

We agree that our model does not explore the precise mineralization mechanisms of different polymers and differences in mineralization based on subunit composition, but this is also not the goal of our model. Please note that the model is heavily based on data from Chasteen and Theil; the values for model parameters k_{cat}, (Oxidation) and k_{deg} (Reduction) are exactly those that were determined in Liu X, Theil EC 2004 (ref 19). The validation data in Figures 2b, 2c and 2f are also from Chasteen and Theil (Tosha 2008, Zhao 2003, Zhao 2005, refs 38, 21, and 41). Also, the data from these authors that we used for calibration and validation have different subunit compositions, from low-H (4H) to full-H homopolymer described in the updated Fig 2 legend.

An Equal Opportunity Employer

Cell and Genome Sciences Building
400 Farmington Avenue
Farmington, CT 06030-6406
PHONE 860.679.1452 · FAX 860.679.1030

Richard D. Berlin
Center for cell analysis and modeling
health.uconn.edu/cell-analysis-modeling/
pmendes@uchc.edu

UCONN HEALTH

Richard D. Berlin Center for
Cell Analysis and Modeling

8. **In general, the authors appear to neglect the fact that several of the better ferritin manuscripts in the literature (incl the 2005 JMB paper cited above) provided a full kinetic analysis, and model the different species that are formed based on UV-Vis spectroscopy. These studies succeed in explaining mineralization kinetics for a specific ferritin heteropolymer, e.g., horse spleen apoferritin. The authors here are somewhat vague about which ferritin they are trying to model, but it seems to be overreaching to try to explain ALL ferritin biochemistry with this simplistic model. The DFP species may vary in its prevalence and mechanistic significance depending on the ferritin composition, etc.**

We could not agree more. The model presented in our manuscript does not attempt to explain all ferritin biochemistry. We are thankful for this comment as it allowed us the opportunity to revise the manuscript where necessary to correct any indication of such tone. We have added clarifications in the discussion on how the model should be used. In contrast to explaining all FT biochemistry, our intent is instead to provide a model that is simple and accessible enough (via community standard file format, SBML, and COPASI format) to be a useful <u>component</u> for incorporation into larger models of <u>cellular</u> iron metabolism and regulation. In some cases the model could be used as is (such as our example with hepatocyte iron metabolism), but in other cases the model will need to be expanded to address specific issues. For example the question raised by reviewer 2 on modeling mitochondrial ferritin is likely to require adaptations in the rate laws to properly represent the functional behavior of that ferritin. Another case would be modeling the interaction with other metals that could be incorporated into the ferritin core, where likely extra reactions would need to be added. In the discussion we comment on these possible expansions.

An Equal Opportunity Employer

Cell and Genome Sciences Building
400 Farmington Avenue
Farmington, CT 06030-6406
PHONE 860.679.1452 · **FAX** 860.679.1030

Richard D. Berlin
Center for cell analysis and modeling
health.uconn.edu/cell-analysis-modeling/
pmendes@uchc.edu
